# Pattern recognition receptors in the gut: analysis of their expression along the intestinal tract and the crypt/villus axis

**DOI:** 10.14814/phy2.12225

**Published:** 2015-02-12

**Authors:** Pascal Gourbeyre, Mustapha Berri, Yannick Lippi, François Meurens, Silvia Vincent-Naulleau, Joëlle Laffitte, Claire Rogel-Gaillard, Philippe Pinton, Isabelle P Oswald

**Affiliations:** 1 INRA, UMR1331, Toxalim, Toxicologie AlimentaireToulouse, France; 2 Université de Toulouse, INP, UMR1331, ToxalimToulouse, France; 3 INRA, UR1282, ISP, Infectiologie et Santé PubliqueNouzilly, France; 4 Université François Rabelais, UMR1282 Infectiologie et Santé PubliqueTours, France; 5 Vaccine and Infectious Disease Organization-International Vaccine Centre (VIDO-InterVac), University of SaskatchewanSaskatoon, Saskatchewan, Canada; 6 INRA, UMR1313, Génétique Animale et Biologie IntégrativeJouy-en-Josas, France; 7 AgroParisTech, UMR1313 Génétique Animale et Biologie IntègrativeJouy-en-Josas, France; 8 CEA, DSV, IRCM, Laboratoire de Radiobiologie et Etude du Génome, Domaine de VilvertJouy-en-Josas, France

**Keywords:** Immune system, NOD, pig, RIG-I, small intestine, TLR

## Abstract

Pattern recognition receptors (PRRs) play a critical role in the detection of microorganisms and the induction of inflammatory and immune responses. Using PCR and Western-blot analysis, this study investigated the differential expression in the intestine of 14 PRRs and nine associated cytokines. Thirty-two pigs were used to determine the expression of these markers (1) along the proximal/distal axis of the small intestine (duodenum, jejunum, and ileum) and (2) between the intestinal segments and their respective lymphoid organs (Peyer's patches [PP] and mesenteric lymph nodes [MLN]). Six additional animals were used to quantify the expression of these genes along the crypt/villus axis of jejunum, using microdissected samples. Most genes showed increased expression (1) in the distal than in the proximal parts of the small intestine (TLR3, 5, RIG-I, IL-1*β*, IL-8, and IFN-*γ*); (2) in lymphoid organs (TLR1, 2, 6, 9, 10, IL-10, TNF-*α*), especially the MLN (TLR4, 7, 8, NOD1, NOD2, NALP3, IFN-*α*, IL-6, IL-12, and TGF-*β*), than in intestinal segments. The analysis along the crypt/villus identified: (1) genes with higher expression in *lamina propria* (TLR1, 2, 4, 9, NOD1, NOD2, IL-1*β*, IL-10, TGF-*β*, TNF-*α*) and (2) genes with higher expression in the villus (TLR3, 5, 6, RIG-I, IL-6). These results highlight the differential expression of PRRs and cytokines along the proximal/distal and the crypt/villus axis of the intestine, contributing to a fine analysis of the complex functional architecture of the small intestine and should be related to the gut microbiota.

## Introduction

The major functions of the small intestine are linked to food digestion with segment-specific activity: the proximal parts of the intestinal tract are involved in the enzymatic cleavage of food molecules and the distal parts in nutrient, vitamin, and salt absorption (Jackson and McLaughlin 2009). Beside digestion, the small intestine is also involved in hormone secretion, immune regulation, and microorganism sensing (Wapnir and Teichberg 2002; Abreu 2010). Pattern recognition receptors (PRRs) are membrane-bound receptors either expressed on cell surfaces or associated with intracellular vesicles that specifically bind to pathogen-associated molecular patterns (PAMPs) shared by various microorganisms. Different families of PRRs have been identified (Mair et al. 2014) including (among other receptors), toll-like receptors (TLRs), Nuclear Oligomerization Domain-like receptors (NLRs), and Retinoic acid Inducible Gene-like receptors (RLRs). TLRs recognize various PAMPs from bacteria (lipoproteins, lipopolysaccharides, flagellin, DNA, single-strand RNA), viruses (structural proteins, double strand RNA, single-strand RNA), fungi (*β*-glucan, zymosan, mannan), and parasites (Kawai and Akira 2001). Some TLRs also bind to nucleic acids, DNA or RNA, shared by all microorganisms (TLR9 and TLR7 recognize DNA and RNA, respectively). NLRs are cytosolic proteins that respond to various PAMPs from bacteria (lipoproteins, pore-forming toxins, RNA), viruses (RNA), fungi, and parasites (Kawai and Akira 2001; Williams et al. 2010). RLRs such as RIG-I or melanoma differentiation-associated protein 5 (MDA5) are RNA helicases binding to double-strand viral RNA.

PRR expression is linked to the sensitivity of the host toward either microorganisms from microbiota or invading pathogens. They are key mediators for the induction of an inflammatory response. For example, TLR signaling activates either the Myeloid Differentiation 88 (MyD88)-dependent pathway leading to nuclear factor kappa B (NFkB) activation, or the TIR-domain-containing adapter-inducing interferon-*β* (TRIF)-dependent pathway leading to interferon regulatory factors 3 (IRF3) and/or IRF7 activation (Kumar et al. 2011). Ligand binding to either NOD1 or NOD2 induces downstream signaling cascades that activate NFkB (Kumar et al. 2011). Nacht domain-, LRR-, and PYD-containing protein 3 (NALP3) and many other NLR act through the cleavage of caspase 1 and the recruitment of inflammasomes (Williams et al. 2010). All RLR described induce either NFkB or IRF3/7 pathways (Kumar et al. 2011). NFkB transcription factor induces the expression of gene coding for inflammatory cytokines such as IL-1*β*, IL-6, IL-8, IFN*γ*, and TNF*α,* whereas IRF3/7 promote the production of type I interferons like IFN*α* and IFN*β* (Kumar et al. 2011). Inflammasomes promote the maturation of IL-1*β* and IL-18 (Williams et al. 2010).

PRRs are important sensors in the crosstalk between microorganisms and the host, and are expressed in various mucosal cell types. PRR expression in immune cells is well described (Werling and Jungi 2003; Alvarez et al. 2008) but expression of these receptors in the intestine is less documented. In humans, some studies have shown that TLR3 and TLR5 are constitutively expressed by intestinal epithelial cells from both colon and ileum and that TLR2 and TLR4 are less expressed by these cell types (Cario and Podolsky 2000; Abreu 2010). The intestine, as a first barrier, is in contact with commensal and pathogenic microorganisms (Abreu 2010). It is important to analyze the expression of PRRs along the small intestine, in local lymphoid organs and along the crypt/villus axis in order to elucidate how this organ senses commensal flora as well as potentially pathogenic microorganisms. Increased knowledge about intestinal expression of PRRs and cytokines associated with these PRRs signaling pathways is important to better understand the regulation of gut innate immunity.

The pig has a small intestine that shares common proportions and histological features with its human counterpart (Swindle et al. 2011). Additionally, the pig innate and adaptive immune systems share common sensors with those of humans (Dawson et al. 2013; Mair et al. 2014). Thus, the pig constitutes an interesting model for human intestine and it is possible to sample large portions of proximal segments. Our aim was to analyze the differential expression of genes along the small intestine of the pig in order to decipher the functional variations according to axis of this organ. A recent study has reported a wide range of commonly, but also differentially, expressed genes along the small intestine and in ileal Peyer's Patches (PP) by transcriptome sequencing (Mach et al. 2014). In this complementary study, we focused on the expression of a comprehensive set of PRRs (TLR1 to 10, NOD1, 2, NALP3, and RIG-I) and genes associated with PRR signaling pathways (IFN*α*, IFN*γ*, IL-1*β*, IL-6, IL-8, IL-10, IL-12, TNF*α,* and TGF*β*) at transcript and protein levels, and investigated variations in gene expression along the proximal/distal axis (duodenum, jejunum, ileum), between small-intestine-related lymphoid organs (jejunal PP, ileal PP, Mesenteric Lymph Nodes: MLN) and their corresponding intestinal segments, and along the crypt/villus axis of jejunum (crypt, villus, and *lamina propria*).

## Materials and Methods

### Animals

Thirty-two 70-day-old Large White pigs with an average weight of 27 kg were used for this study. All animals were bred in the experimental farm unit of Le Magneraud (France). Animals were fed ad libitum. All animal experiments were carried out in accordance with European Guidelines for the Care and Use of Animals for Research Purposes. The animal protocol was approved by the local ethics committee COMETHEA and assigned the agreement number CE2013-2.

### Sample collection

Following euthanasia, samples from duodenum, jejunum, ileum, jejunal PP, ileal Peyer's Patches, and MLN were collected. Duodenum sample was taken at 30 cm away from the stomach. Ileum was sampled at 30 cm before the ileocecal junction. Full thickness sections of the tissue including epithelium, submucosal, and muscularis were cut in small pieces of 0.5 cm^2^, snap-frozen in liquid nitrogen and stored at −80°C until further use.

### Real-time quantitative PCR (qPCR)

Duodenum, jejunum, ileum, PP from jejunum and ileum, and MLN samples from 32 pigs were lysed in 1 mL of Trizol reagent (Invitrogen, Cergy Pontoise, France) with ceramic beads (Bertin technologies, St Quentin en Yvelines, France). Total RNA was purified using RNeasy Mini Kit (Qiagen, Courtaboeuf, France) according to the manufacturer's recommendations. Residual genomic DNA was removed using DNase digestion with RNase-free DNase I Amp Grade (Invitrogen) (1 U/*μ*g of RNA) following the recommended protocol. RNA concentration was determined by measuring optical density at 260 nm (OD260), and the RNA integrity was assessed by using both a NanoDrop spectrophotometric analysis (NanoDrop Technologies, Wilmington, NC) and Agilent capillary electrophoresis (Agilent 2100 Bioanalyzer, Agilent Technologies Inc., Santa-Clara, CA). All samples had an RNA integrity number above 8, which is indicative of high quality RNA. Total RNA samples were reverse-transcribed using High Capacity cDNA-RT kit (Life Technologies, Saint Aubin, France). Real-time quantitative PCR were performed in 384-well plates in a ViiA 7 thermocycler (Life Technologies) as already described (Pié et al. 2007; Cano et al. 2013). Primers for the real-time quantitative PCR are presented in Table1. Cyclophilin A and RPL-32 genes were chosen as internal reference genes for data normalization (Cano et al. 2013). Real-time PCR values are expressed as absolute abundance/gene copy number (delta Ct method).

**Table 1:** Primers used for real-time quantitative PCR analysis

Gene symbol	Gene name	Primer sequence	mRNA ID	References
ALP	Alkaline phosphatase	F: AAGCTCCGTTTTTGGCCTG	ENSSSCT00000017732	Current article
		R: GGAGGTATATGGCTTGAGATCCA		
Cyclo A	Cyclophilin A	F: CCCACCGTCTTCTTCGACAT	NM_214353	Curtis and Way (2009))
		R: TCTGCTGTCTTTGGAACTTTGTCT		
EDN2	Endothelin 2	F: TACTTCTGCCACTTGGACATCATC	ENST00000372587	Current article
		R: GGCCGTAAGGAGCTGTCTGT		
IFNα	Interferon alpha	F: ATGAGATGCTCCAGCAGAC		Current article
		R: TTTCCTCACAGCCAGGATG		
IFNγ	Interferon gamma	F: TGGTAGCTCTGGGAAACTGAATG	NM_213948	Current article
		R: GGCTTTGCGCTGGATCTG		
IL-1β	Interleukine-1 beta	F: ATGCTGAAGGCTCTCCACCTC	NM_214055	von der Hardt et al. (2004)
		R: TTGTTGCTATCATCTCCTTGCAC		
IL-6	Interleukine-6	F: TTCACCTCTCCGGACAAAACTG	NM_214399	Current article
		R: TCTGCCAGTACCTCCTTGCTGT		
IL-8	Interleukine-8	F: GCTCTCTGTGAGGCTGCAGTTC	NM_213867	Bracarense et al. (2012)
		R: AAGGTGTGGAATGCGTATTTATGC		
IL-10	Interleukine-10	F: GGCCCAGTGAAGAGTTTCTTTC	NM_214041	Bracarense et al. (2012)
		R: CAACAAGTCGCCCATCTGGT		
IL12-p40	Interleukine-12 p40	F: GGTTTCAGACCCGACGAACTCT	NM_214013	Cano et al. (2013)
		R: CATATGGCCACAATGGGAGATG		
Lysozyme	Lysozyme	F: GGTCTATGATCGGTGCGAGTTC	ENSSSCT00000034939	Current article
		R: TCCATGCCAGACTTTTTCAGAAT		
NALP3	NACHT, LRR and PYD domains-containing protein 3	F: AGCTAAGAGGGATGAGCCAGAA	ENSSSCT00000015226	Current article
		R: CTTATCACAGAAAGATTTGCATTGTCT		
NOD1	Nuclear oligomerization domain 1	F: TGGGCTGCGTCCTGTTCA	AB_187219,1	Mariani et al. (2009)
		R: GGTGACCCTGACCGATGT		
NOD2	Nuclear oligomerization domain 2	F: GAG CG CATCCTCTTAACTTTC		Meurens et al. (2009)
		R: ACGCTCGTGATCCGTGAAC		
PCNA	Proliferating cell nuclear antigen	F: GTTGATAAAGAGGAGGAAGCAGTT	ENSSSCT00000032581	Current article
		R: TGGCTTTTGTAAAGAAGTTCAGGTAC		
RIG-I	Retinoic acid-inducible gene 1	F: CCACCTTCATCCTGAGCTACATG	ENSSSCT00000026775	Current article
		R: TTGTTTTTCTCAGCCTGAATATGC		
RPL 32	60S ribosomal protein L32	F: AGTTCATCCGGCACCAGTCA	NM_001001636	Pinton et al. (2010)
		R: GAACCTTCTCCGCACCCTGT		
TGFβ	Transforming growth factor beta	F: GAAGCGCATCGAGGCCATTC		Levast et al. (2010)
		R: GGCTCCGGTTCGACACTTTC		
TLR1	Toll-like receptor 1	F: TGCTGGATGCTAACGGATGTC	AB219564	Arce et al. (2010)
		R: AAGTGGTTTCAATGTTGTTCAAAGTC		
TLR2	Toll-like receptor 2	F: TCACTTGTCTAACTTATCATCCTCTTG	AB085935	Arce et al. (2010)
		R: TCAGCGAAGGTGTCATTATTGC		
TLR3	Toll-like receptor 3	F: AGTAAATGAATCACCCTGCCTAGCA	DQ266435	Arce et al. (2010)
		R: GCCGTTGACAAAACACATAAGGACT		
TLR4	Toll-like receptor 4	F: GCCATCGCTGCTAACATCATC	AB188301	Arce et al. (2010)
		R: CTCATACTCAAAGATACACCATCGG		
TLR5	Toll-like receptor 5	F: CCTTCCTGCTTCTTTGATGG	NM_001123202	Meurens et al. (2009)
		R: CTGTGACCGTCCTGATGTAG		
TLR6	Toll-like receptor 6	F: AACCTACTGTCATAAGCCTTCATTC	AB085936	Arce et al. (2010)
		R: GTCTACCACAAATTCACTTTCTTCAG		
TLR7	Toll-like receptor 7	F: CAGAAGTCCAAGTTTTTCCAGCTT		Current article
		R: GGTGAGCCTGTGGATTTGTTG		
TLR8	Toll-like receptor 8	F: AAGACCACCACCAACTTAGCC	AB092975	Arce et al. (2010)
		R: GACCCTCAGATTCTCATCCATCC		
TLR9	Toll-like receptor 9	F: CACGACAG CCGAATAGCAC	AY859728	Arce et al. (2010)
		R: GGGAACAGGGAGCAGAGC		
TLR10	Toll-like receptor 10	F: CTTTGATCTGCCCTGGTATCTCA	AB_208699,1	Current article
		R: CATGTCCGTGCCCACTGAC		
TNFα	Tumor necrosis factor alpha	F: ACTGCACTTCGAGGTTATCGG	NM_214022	Meissonnier et al. (2008)
		R: GGCGACGGGCTTATCTGA		

### Fluorescent Western blotting

Ileum, ileal Peyer's Patches, and MLN samples of 0.5 cm^2^ from eight 70-day-old pigs were homogenized in lysis buffer (20 mmol/L Tris, pH 8.0, 5 mmol/L EDTA, 1% Triton X-100, 0.02% sodium azide, and a protease inhibitor cocktail [Roche, Bale, Switzerland]). The protein concentrations were quantified in duplicate with a colorimetric assay (Bio-Rad Laboratories, Munich, Germany) using albumin as the protein standard. The electrophoresis was performed using a 12% SDS–polyacrylamide gel. The separated proteins were electrotransferred onto nitrocellulose membranes (GE Healthcare, Life Sciences, Piscataway, NJ). Before immunostaining, the nonspecific binding sites were blocked with 5% blocking buffer for fluorescent Western blotting (Rockland Tebu-bio MB-070-010) in Tris-buffered saline (TBS) containing 0.1% Tween-20. The membranes were incubated for 1 h with the respective 1:500 diluted detection antibodies specific for: *β*-actin; RIG-I, TLR7, and TLR9 (Cell Signaling, Leiden, The Netherlands). After several washes in TBS containing 0.1% Tween-20, the membranes were incubated with the appropriate secondary antibodies conjugated with a fluorochrome (CF770 goat anti-rabbit IgG 20078 or CF680 goat anti-rabbit IgG 20067, Biotium, Hayward, CA), which were diluted to 1:5000. The fluorescence was measured at 700 nm (red) and 800 nm (green) with an Odyssey imaging system (Odyssey-Li-COR-Bioscience, Lincoln, NE). The protein expression was estimated as previously described (Pinton et al. 2012).

### Laser microdissection

Six additional animals were used. Jejunum fragments were sampled from six 70-day-old pigs. Jejunum samples were opened longitudinally to expose the inner structures, covered with a thin layer of embedding medium (Tissue-Tek®), rolled on a toothpick (Swiss roll technique), and directly cooled in isopentane previously cooled in liquid nitrogen. Sequentially, the roll was placed in a container, surrounded by Tissue-Tek medium and then frozen in liquid nitrogen for later cryodissection.

Twelve-*μ*m-thick sections were produced at −20°C from embedded fragments using a cryostat at −20°C. Tissues sections were first fixed with cold ethanol (55%; −20°C; Fluka, St Quentin Fallavier, France) for 30 sec, soaked in ethanol (50%) for 20 sec and stained with Cresyl violet (Ambion, Villebon sur Yvette, France) for 30 sec. Tissue sections were further dipped in 50% ethanol for 20 sec and finally dipped consecutively in a gradient of ethanol baths. Lastly slides were soaked twice in 100% anhydrous Xylene (Sigma-Aldrich, Lyon, France) for 5 min each, then air-dried and kept in anaerobic conditions until microdissection.

Dyed slides were placed in the microdissector (Arcturus XT™, Life Technology) with the magnification set at 25X. The laser gently heats the adhesive layer on the cap's bottom (CapSure macroLCM caps Excilone, Elancourt, France) fusing it to the underlying tissue (villus epithelium, crypt epithelium, or *Lamina propria*) and allowing the extraction of cells from the slide. Selected tissues were blocked with lysis buffer containing *β*-mercaptoethanol (Qiagen). The microdissection process was limited to 2 h for each slide in order to prevent RNA degradation. Total RNAs were then extracted using the RNeasy Micro Kit (Qiagen).

### Statistical analysis

Data were processed using R packages FactoMineR (www.r-project.org) for Principal Component Analysis (PCA) and Limma, gplots, and marray for heatmap analysis. For data following a normal distribution (D'Agostino & Pearson omnibus normality test), analyses of variance (ANOVA) and Student's t tests were performed. For data not following a normal distribution, the Kruskall–Wallis and Mann–Whitney tests were used. *P*-values below 0.05 (*P* ≤ 0.05) were considered significant.

## Results

### PRRs and genes associated with their signaling pathways are differentially expressed along the intestinal segments and gut lymphoid organs

Expression of 14 PRRs genes (TLR1 to 10, NOD1, NOD2, NALP3, RIG-I) was first studied along the small intestine (duodenum, jejunum, and ileum) and in the corresponding lymphoid organs (jejunal PP, ileal PP, and MLN). Cytokines genes associated with PRR signaling pathways and involved in inflammatory responses (IFN-*α*, IFN-*γ*, IL-1*β*, IL-6, IL-8, IL-12, and TNF-*α*) as well as cytokines implicated in their retro-control (IL-10 and TGF-*β*) were included in this study.

In the case of gene expression along the proximal–distal axis, most tested genes were less expressed in duodenum than in distal segments: that was the case for TLR1, 2, 3, 4, 5, 6, 8, 9, 10, NOD1, 2, RIG-I, IL-1*β*, IL-6, IL-10, IL-12, TGF-*β*, TNF-*α*, and IFN-*γ* (Table2 and Fig. 2). A few genes, such as TLR1, 2, 4, and IL-6 also showed a gradual increase in expression from duodenum to ileum (Table2 and Fig. 2). Along the same axis, several tested genes were differentially expressed between PP from the jejunum and the ileum. TLR2, 6, 9, 10 showed increased expression in the ileal PP, whereas TLR7, NALP3, IL-1*β*, IL-8, IL-12, TNF-*α*, and IFN-*γ*, were more expressed in the jejunal PP (Table2 and Fig. 2B).

**Table 2:** Transcript expression of genes tested in the current study in duodenum, jejunum, ileum, PP from jejunum, PP from ileum and MLN from 32 pigs. Only genes that were not plotted on Figure2B were included in this table. Values are expressed as absolute abundance/gene copy number (delta Ct method) and normalized using duodenum as control. According to the normality of the data distribution, either ANOVA test followed by Student's t-test, or Kruskall–Wallis test followed by Mann–Whitney test were used to calculate *P* values. Values with no common superscripts are significantly different (*P* < 0.05). SD, standard deviation

	Duodenum	Jejunum	Ileum	Peyer jejunum	Peyer ileum	MLN
TLR2						
Mean	1.00^a^	2.24^b^	23.27^d,e^	8.44^c^	16.52^d^	28.79^e^
SD	1.13	1.36	15.61	5.40	11.30	8.52
TLR4						
Mean	1.00^a^	1.45^b^	2.09^c^	1.95^c^	2.02^c^	5.14^d^
SD	0.60	0.58	0.94	0.74	1.18	2.18
TLR5						
Mean	1.00^a^	2.66^b^	2.92^b^	1.42^c^	1.92^c^	1.17^c^
SD	1.21	1.73	2.10	0.80	2.14	0.57
TLR6						
Mean	1.00^a^	2.06^b,c^	2.15^b,c^	1.66^b^	2.42^c^	2.82^d^
SD	0.48	1.00	1.19	0.79	1.24	0.88
TLR8						
Mean	1.00^a^	2.55^b^	1.47^c^	2.63^b^	2.12^b,c^	9.36^d^
SD	0.79	1.93	0.99	1.99	1.80	4.85
TLR10						
Mean	1.00^a^	1.70^b^	3.59^b^	6.53^c^	9.46^d^	12.72^e^
SD	0.52	1.30	3.92	3.71	4.66	3.66
NOD1						
Mean	1.00^a^	3.37^b^	3.88^b^	5.53^c^	4.57^b^	24.46^d^
SD	1.67	3.18	3.02	3.10	5.45	12.99
NALP3						
Mean	1.00^a^	1.08^a^	0.70^b^	0.90^c^	0.72^b^	1.59^d^
SD	0.50	0.21	0.35	0.30	0.36	0.78
IL-8						
Mean	1.00^a,b^	1.07^a^	1.03^a,b^	0.83^b^	0.51^c^	0.06^d^
SD	0.48	0.37	0.49	0.48	0.43	0.07
IL-10						
Mean	1.00^a^	2.64^b^	2.99^b,c^	3.02^b,c^	3.25^c^	4.42^d^
SD	0.70	1.46	1.50	1.16	0.87	1.94
IL-12						
Mean	1.00^a^	2.68^b^	1.96^b^	4.19^c^	2.10^b^	15.43^d^
SD	0.89	1.84	1.62	2.16	1.58	11.06
IFN alpha						
Mean	1.00^a^	1.19^a,b^	1.44^a,b^	1.60^b^	1.18^a,b^	3.33^c^
SD	0.65	0.71	0.80	1.13	0.76	1.66
IFN gamma						
Mean	1.00^a^	1.85^b,c^	2.48^b^	1.72^c^	1.49^a,d^	1.43^d^
SD	0.55	0.84	1.73	0.84	1.63	0.65
TGF beta						
Mean	1.00^a^	2.75^b^	2.53^b^	3.94^c^	3.99^c^	8.91^d^
SD	0.84	0.96	1.13	1.45	1.61	2.99

**Figure 1:**
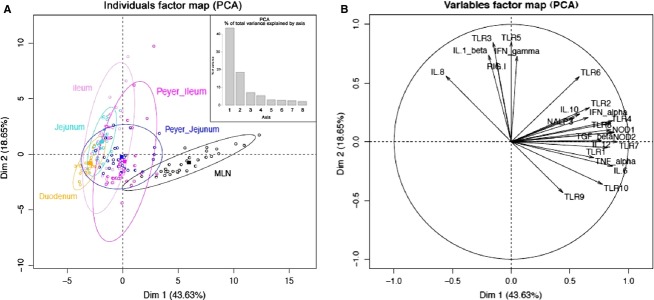
Principal component analysis of PRR expression along the proximal–distal axis and between segments and lymphoid organs. Individuals factor map (A) and variable factor map (B). On the upper right hand corner of individual factor map the bar plot correspond to PCA percentage of total variance explained by axis.

**Figure 2:**
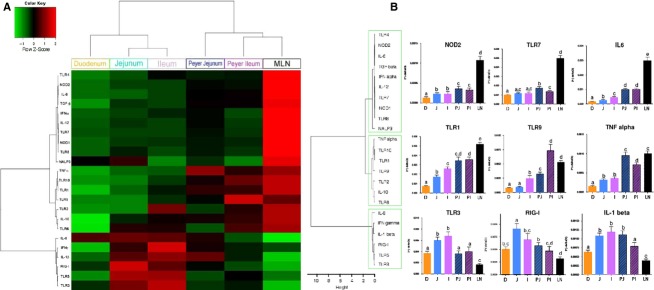
Heatmap (A) and cluster (B) analyses of PRR expression along the proximal–distal axis and between segments and lymphoid organs from 32 pigs. Duodenum (D) is plotted in orange, jejunum (J) in light blue, ileum (I) in light violet, Peyer's Patches from jejunum (PJ) in either blue for PCA or light blue hatched for bar plots, Peyer's Patches from ileum (PI) in either violet for PCA or light violet hatched for bar plots, and MLN (LN) in black. Figure2B illustrates the expression levels of three genes belonging to each group. Bar plots within each graph with no common superscripts are significantly different (*P* < 0.05). The three gene groups are indicated on the left of the figure in the green line boxes. Data are expressed as means ± SEM.

A PCA (multidimensional scaling analysis) (Fig.1) and a heatmap analysis (Fig.2) were carried out based on the expression of the set of 23 normalized genes included in our study in the six intestinal organs from 32 animals. PCA analysis allows finding structure in data by rescaling a set of dissimilarity measurement into distances assigned to specific locations in a special configuration. The individual factor map (Fig.1A) shows that MLN samples from all the 32 pigs used for this experiment are deviated to the right of the plot. This pattern is due to MLN expression of all genes plotted on the right of variable factor map (all vectors that point to the right in the Fig.1B). The heatmap technique gives a hierarchy and a graphical representation of a large set of data where all individuals’ values are plotted as colors. The heatmap plot (Fig.2A) also shows that MLN display a highly distinct pattern compared with the five other parts of the intestine. Both analyses distinguish intestinal segments from their corresponding lymphoid organs (jejunum vs. jejunal PP and ileum vs. ileal PP) (Figs.1A, 2B). PCA and heatmap hierarchy analyses enabled us to distinguish three groups of genes (Fig.2B).

The first group of genes includes TLR4, 7, 8, NOD1, NOD2, NALP3, IFN-*α*, IL-6, IL-12, and TGF-*β* (Fig.2A and B). All these genes were more expressed in MLN than in other tissues. Among these genes, TLR4, 7, NOD1, 2, IL-6, IL-12, and TGF- *β* had higher expression in jejunal PP than in jejunum itself. NOD2, IL-6, and TGF-*β* were also more expressed in ileal PP than in ileal tissue surrounding the PP.

The second group of genes included TLR1, 2, 6, 9, 10, IL-10, and TNF-*α* (Fig.2A and B). These genes showed higher expression in lymphoid organs, *i. e*. jejunal or ileal PP and MLN than in the duodenum, the jejunum or the ileum. Among genes belonging to second group, TLR1, 2, 9, 10, and TNF-*α* were more expressed in the jejunal PP than in the jejunum itself and TLR1, 9, 10, and TNF-*α* were also more expressed in PP from ileum than in ileum itself.

The third group of genes includes TLR3, 5, RIG-I, IL-1*β*, IL-8, and IFN-*γ* (Fig.2A and B) with these genes being more expressed in the jejunum and the ileum than in the other organs.

**Figure 3:**
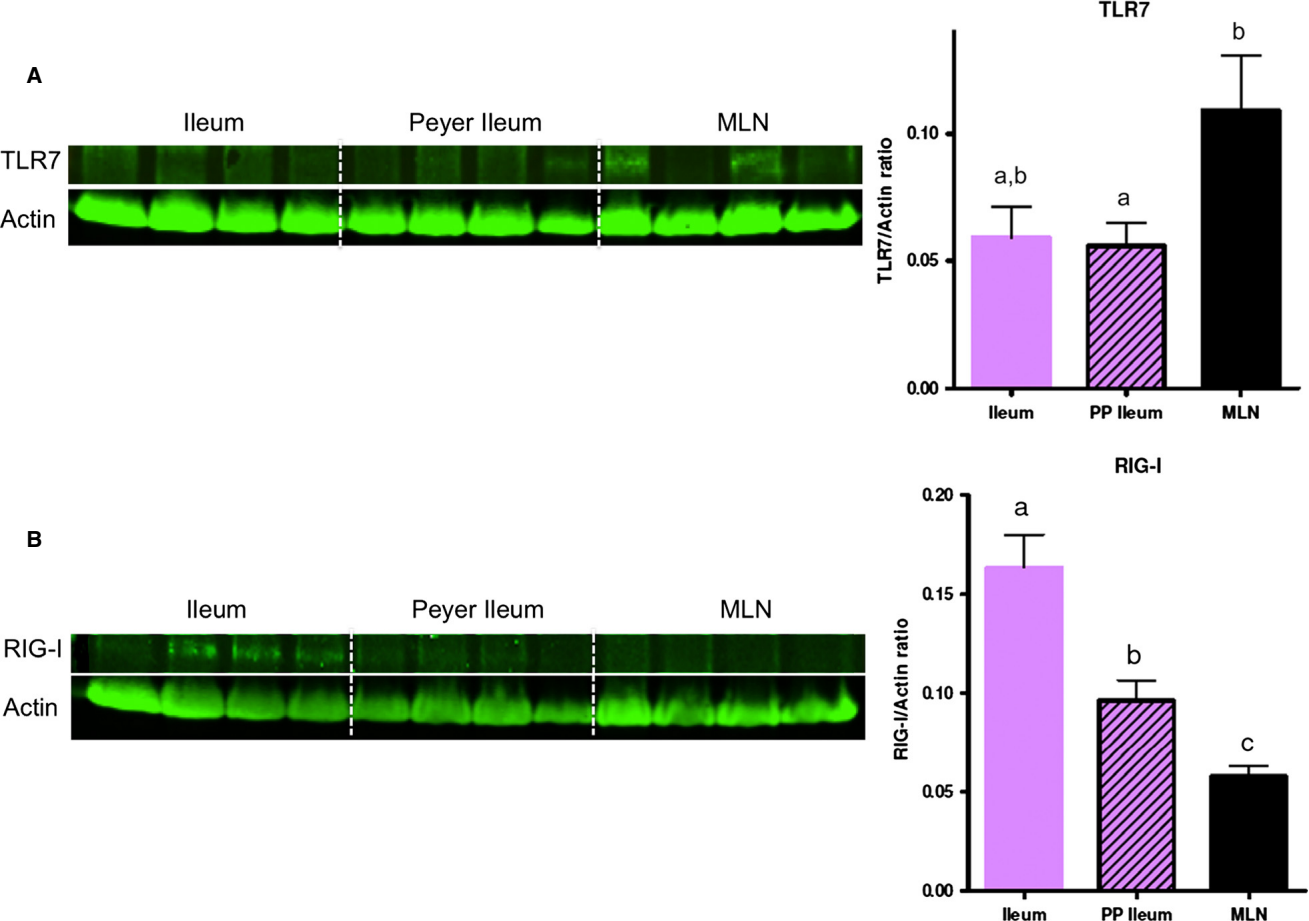
Protein expression of TLR7 (A) and RIG-I (B) in three samples: ileum (light violet), PP from ileum (light violet hatched) and MLN (black) of eight pigs. Only four samples from the eight obtained are on the Figure. Data are expressed as means ± SEM. Bar plots within each graph with no common superscripts are significantly different (*P* < 0.05).

In order to validate the results obtained at the mRNA level, we investigated the protein expression level of three selected PRRs: TLR7, TLR9, and RIG-I in the MLN, the ileal PP and the ileum of eight randomly chosen piglets (Fig.3). Genes were chosen as corresponding to the three groups of gene identified using qPCR approach. TLR7, 9, and RIG-I were selected because the antibody used to detect them displayed the best effectiveness. As already observed at the mRNA level, TLR7 protein was significantly more expressed in MLN than in the ileal PP or in the ileum. However, due to the limited number of animals, the difference between the TLR7 expression in the MLN and the ileum (+ 84%) was not significant (Fig.3A). For RIG-I, Western blot and qPCR analysis showed the same ranking and even with only eight animals, Western blot analysis was more discriminating: RIG-I protein was significantly less expressed in MLN than in the ileal PP and even less in the ileum (Fig.3B). TLR9 protein had a highly variable expression and no significant difference was observed between the three organs tested (data not shown). For TLR9 no difference in protein expression was detected between ileal tissue and the lymphoid structures.

In summary, PRRs and their associated immune response mediators are differentially expressed along the small intestine. Most of these proteins and their transcripts were more expressed in the distal parts (jejunum, ileum) than in duodenum. A differential expression was also observed between lymphoid and nonlymphoid segments (MLN and PP vs. duodenum, jejunum, and ileum).

### PRRs are differentially expressed along the crypt/villus axis

The next objective of this study was to characterize PRR expression along the crypt/villus axis. The jejunum segment from six pigs was microdissected to separate the villus epithelium, the crypt epithelium and the *Lamina propria*.

**Figure 4:**
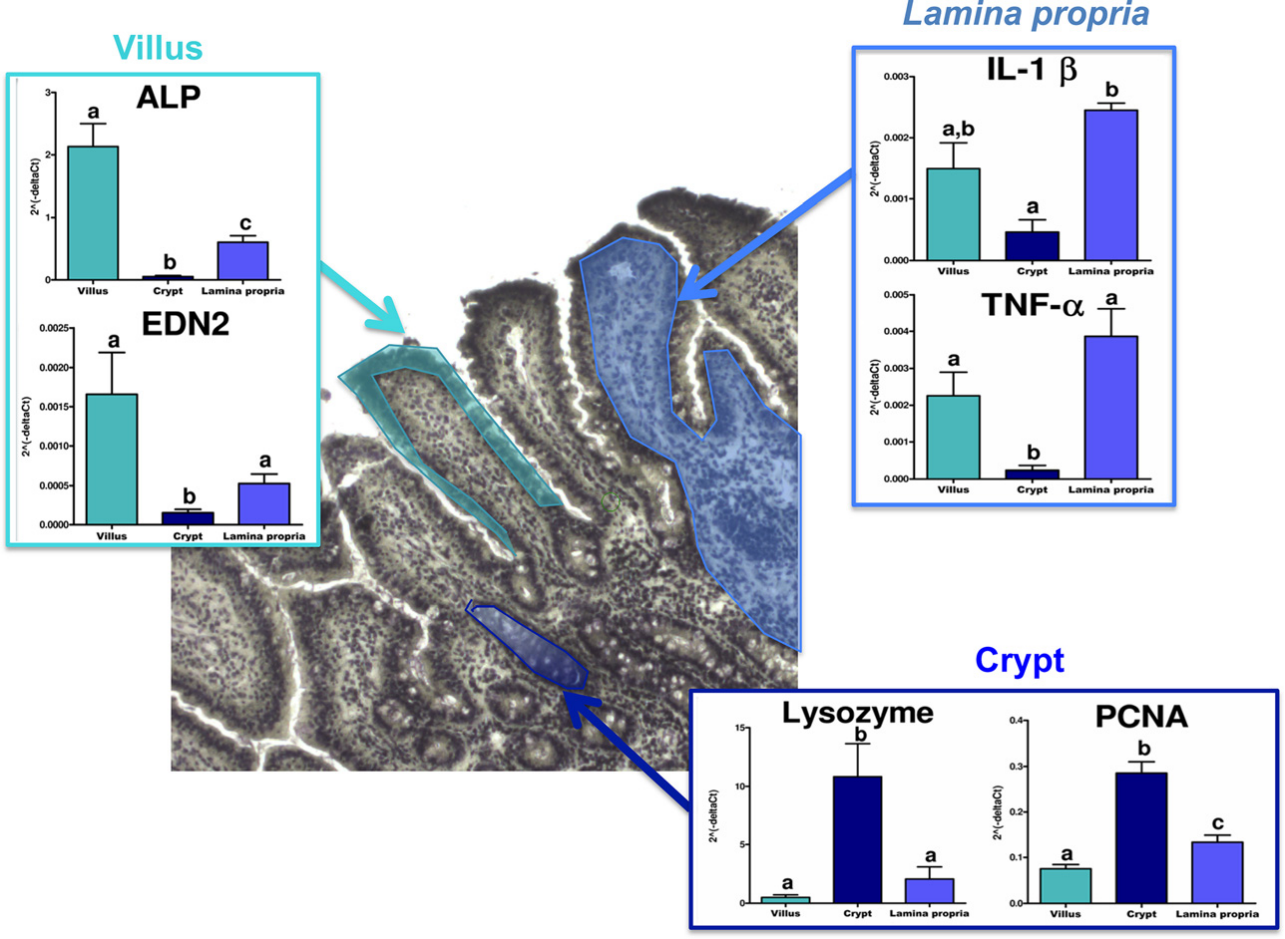
Validation of the laser microdissection technique. Bar plots represent the transcriptomic expression of each gene in either villi (cyan), *Lamina propria* (blue) or crypts (dark blue) from jejunum of six pigs. Data are expressed as means ± SEM. Kruskall–Wallis test followed by Mann–Whitney test was used to calculate *P* values. Bar plots within each graph with no common superscripts are significantly different (*P* < 0.05).

The microdissection technique was firstly validated through the expression of histological structure specific markers: alkaline phosphatase and endothelin-2 only expressed in the villi; IL-1*β* and TNF*α* that are more expressed in the *Lamina propria*; lysozyme and proliferating cell nuclear antigen only expressed in the crypts. The results plotted on Figure4 show that each marker was specifically expressed in its relative histological structure, thus validating the technique used.

**Figure 5:**
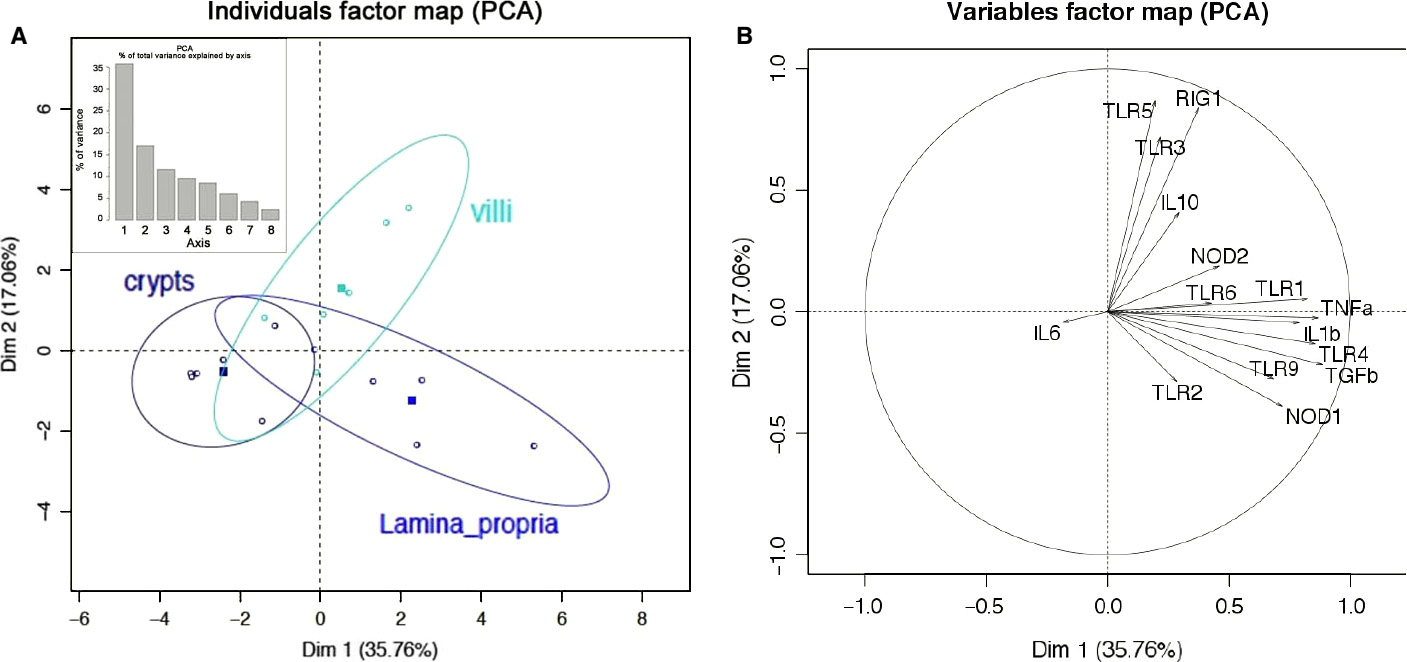
Principal component analysis of PRR expression along the crypt–villus axis. (A) Individuals factor map (PCA) and (B) Variable factor map. On the upper left hand corner of the individual factor map, the bar plot correspond to PCA percentage of total variance explained by axis.

**Figure 6:**
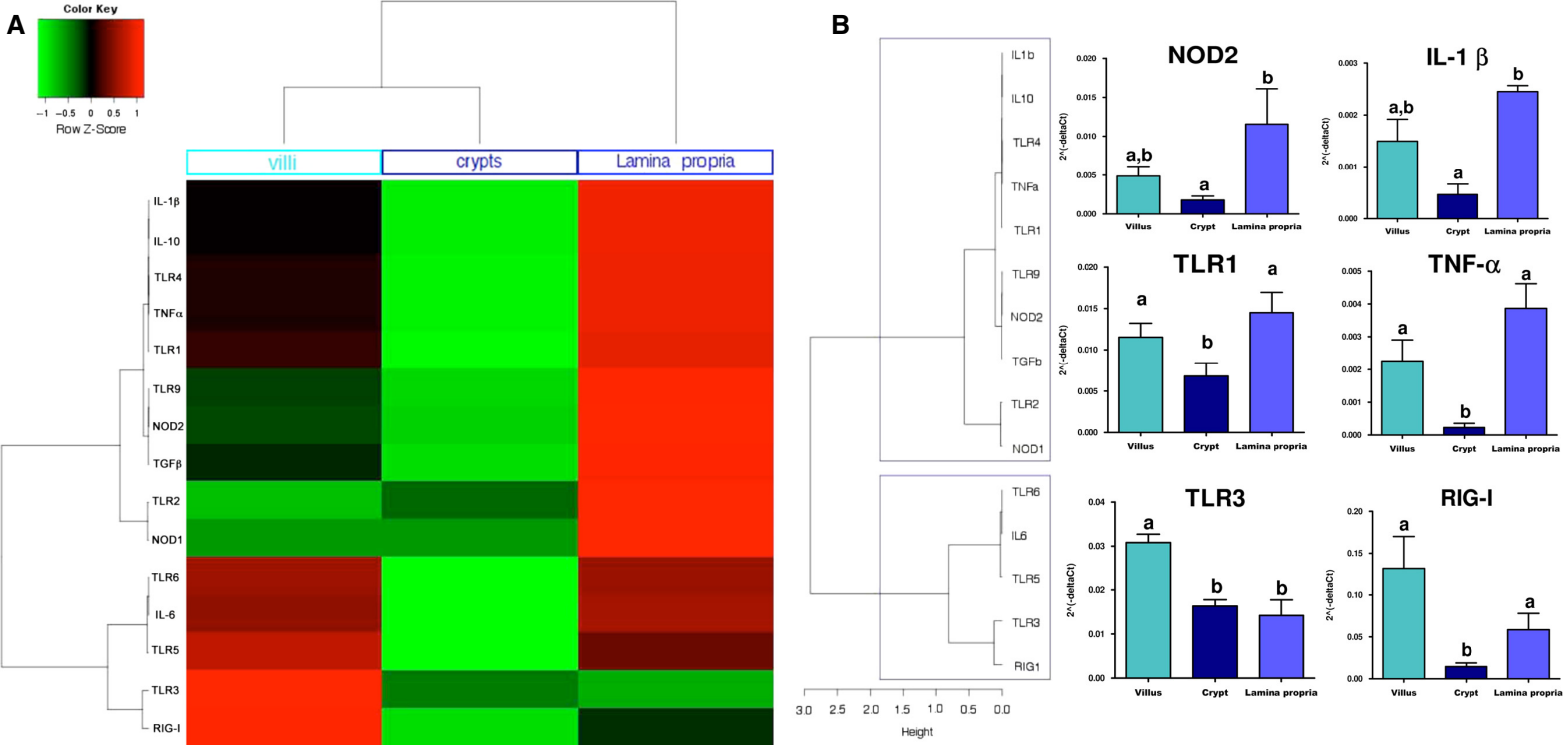
Heatmap (A) and cluster (B) analyses of PRR expression along the crypt–villus axis of the jejunum in six pigs. Figure6B illustrates the expression levels of two genes belonging to each group. The two gene groups are indicated on the left of the figure in the gray line boxes. Data are expressed as means ± SEM. The Kruskall–Wallis test followed by Mann–Whitney test was used to calculate *P* values. Bar plots within each graph with no common superscripts are significantly different (*P* < 0.05).

In a second step, PRR expression was studied in jejunum along the crypt/villus axis in order to delineate the expression of the genes in the villi epithelium, the crypt epithelium and the *Lamina propria*. Due to the small amount of RNA extracted from microdissected arrays, the analysis focused on the 15 genes that were highly expressed in the intestine namely: TLR1, 2, 3, 4, 5, 6, 9, NOD1, NOD2, RIG-I, IL-1*β*, IL-6, IL-10, TGF-*β*, and TNF-*α* (Table3 and Fig. 6B). A PCA (Fig.5) and heatmap (Fig.6) analyses were performed on the qPCR data obtained from the three jejunal structures (villus, crypt and *Lamina propria*) of the six pigs. PCA analysis showed a clear separation of the three histological structures (Fig.5A) and the heatmap enabled two groups of genes to be distinguished (Fig.6B).

**Table 3:** Transcript expression of genes tested in this study in the epithelium of a jejunal villus, *Lamina propria* and epithelium of a jejunal crypt from six pigs. Only genes that were not plotted on Figure6B were included in this table. Values are expressed as absolute abundance/gene copy number (delta Ct method) and normalized using crypt as control. The Kruskall–Wallis test followed by Mann–Whitney test was used to calculate *P* values. Values with no common superscripts are significantly different (*P* < 0.05). SD, standard deviation

	Villus	Crypt	Lamina
TLR2			
Mean	0.77^a^	1.00^a^	1.72^a^
SD	0.29	0.89	1.38
TLR4			
Mean	4.74^a^	1.00^a^	6.83^b^
SD	2.08	1.24	2.59
TLR5			
Mean	1.92^a^	1.00^a^	1.25^a^
SD	0.82	0.45	0.76
TLR6			
Mean	1.46^a^	1.00^a^	1.29^a^
SD	0.63	0.39	0.34
TLR9			
Mean	2.12^a,b^	1.00^a^	2.68^b^
SD	1.27	0.39	1.27
NOD1			
Mean	0.98^a^	1.00^a^	4.37^a^
SD	0.86	1.15	3.60
NOD2			
Mean	2.73^a,b^	1.00^a^	4.24^b^
SD	1.56	0.65	3.39
IL6			
Mean	1.56^a^	1.00^a^	1.90^a^
SD	2.38	1.11	2.42
IL10			
Mean	3.80^a^	1.00^a^	3.77^a^
SD	2.41	0.19	1.41
TGFb			
Mean	4.64^a^	1.00^b^	8.79^c^
SD	0.98	0.82	3.00
ALP			
Mean	41.03^a^	1.00^b^	10.79^c^
SD	15.77	0.86	5.13
EDN2			
Mean	10.88^a^	1.00^a^	3.36^b^
SD	8.57	0.65	2.03
Lysozyme			
Mean	0.05^a^	1.00^b^	0.22^a^
SD	0.05	0.64	0.24
PCNA			
Mean	0.26^a^	1.00^b^	0.46^c^
SD	0.08	0.22	0.15

The first group of genes comprises TLR1, 2, 4, 9, NOD1, NOD2, IL-1*β*, IL-10, TGF-*β*, and TNF-*α*. These genes were mostly expressed in the *Lamina propria* (Table3 and Fig.6B). The second group of genes includes TLR3, 5, 6, RIG-I, and IL-6. Among these genes TLR3 and RIG-I were mostly expressed in villi (Fig.6B). In contrast, TLR5, TLR6, and IL-6 were expressed at the same level in the villi and the *Lamina propria* (Table3). Heatmap analysis also underlines the low expression (cycle threshold ≥35) of all the tested genes (PRRs and cytokines) observed in the crypts (Fig.6A).

Taken together these results show that PRRs and genes associated with their signaling pathways are not uniformly expressed along the crypt/villus axis. Most of these genes are poorly expressed in the crypt epithelium and highly expressed in both the villus epithelium and in the *Lamina propria*.

## Discussion

**Figure 7:**
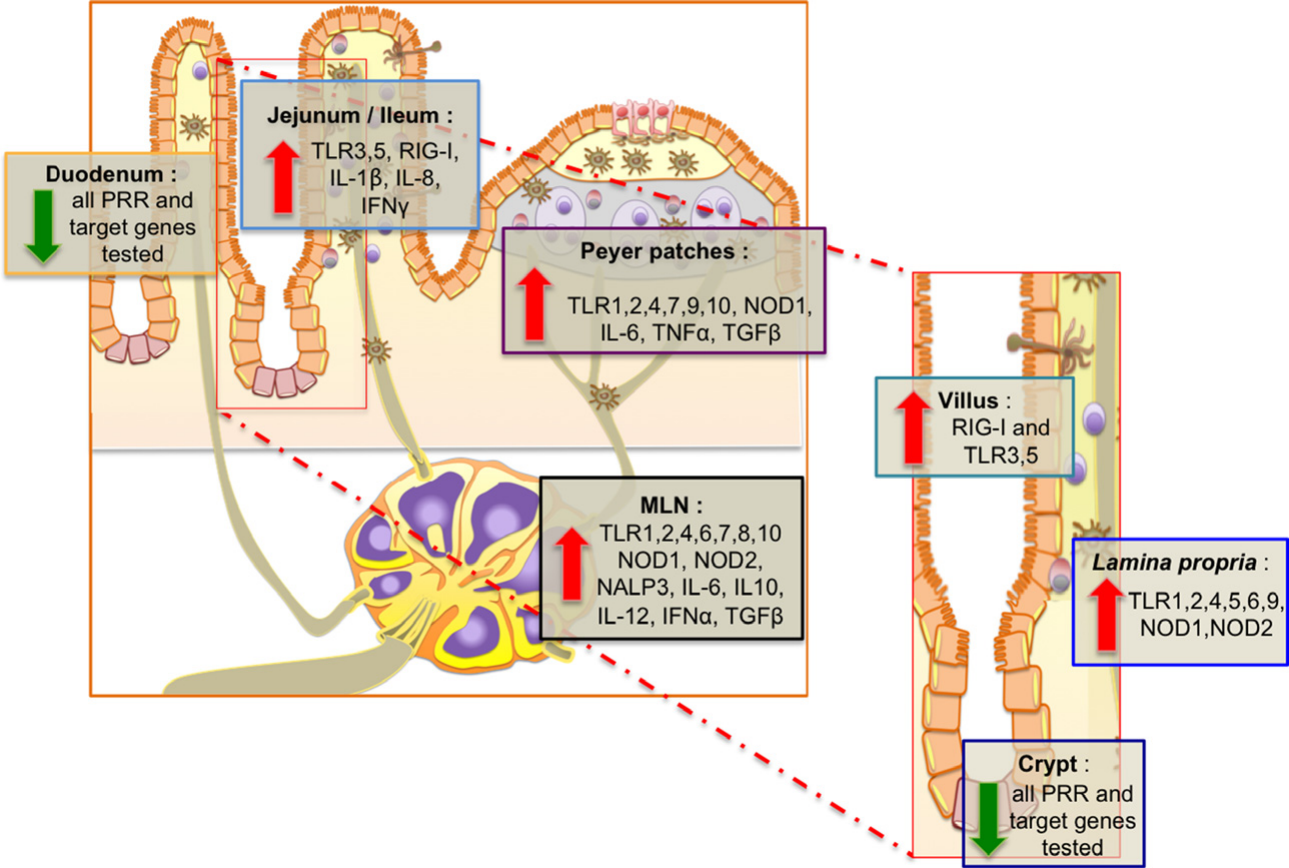
Conclusion graphic. Differential expression of 14 PRRs (TLR 1-10, NOD 1-2, NALP3, and RIG-I) and 9 associated cytokines (IFN*α*, IFN*γ*, IL-1*β*, IL-6, IL-8, IL-10, IL-12, TNF*α,* and TGF*β*) along the proximal/distal axis of the small intestine (duodenum, jejunum, ileum their respective lymphoid organs, and mesenteric lymph nodes) and along the crypt/villus axis (crypt, villus, and *Lamina propria*). Each group of genes highly expressed at the different locations are indicated.

Among its numerous functions, the intestinal tract is an important organ for the defense against ingested microbial pathogens. Through the recognition of the conserved molecular motifs of microorganisms, PRRs are key sensors for maintaining tolerance to commensal microbiota, as well as inducing inflammation against pathogens (Kumar et al. 2011). This study investigated in the small intestine, the differential expression of 14 PRRs (10 TLR, 3 NLR, and RIG-I) as well as nine cytokines associated with these PRRs signaling pathways. As summarized in the conclusion graphic (Fig.7), our data demonstrate that PRRs and the cytokine mRNA are differentially expressed both along the proximal–distal axis and along the crypt–villus axis. To the best of our knowledge, this is the first comprehensive investigation of the expression of these molecules in the small intestine. Indeed, fragmentary data describe the expression of TLRs and NLRs in the porcine intestine (Alvarez et al. 2008; Tohno et al. 2008a,b; Uddin et al. 2013), bovine intestine (Charavaryamath et al. 2011), in rodents (Ortega-Cava et al. 2003; Rumio et al. 2004; Choi et al. 2010; Kosik-Bogacka et al. 2012), and humans (Cario and Podolsky 2000; Otte et al. 2004; Abreu 2010). A few studies have investigated the expression of 10 TLRs in the intestine of either monogastric mammals in relation to the age of the pigs (Uddin et al. 2013), or in ruminants (Menzies and Ingham 2006; Taylor et al. 2008).

### Lower expression of PRRs in the duodenum than in the more distal parts

In mice, data obtained by Ortega-Cava et al. (2003) demonstrated that TLR2 and 4 are less expressed in duodenum than in the distal parts of intestine. Our data extend the lower expression of PRRs in the duodenum to TLR1 to 10, NOD1, NOD2, and RIG-I. A reduced expression of IFN*γ*, IL-1*β*, IL-6, IL-10, IL-12, TNF*α*, and TGF*β* was also observed in this proximal part of the intestine. This weak expression of PRR may be in link with duodenum poor microbiota (Wang et al. 2005). This suggests that the first part of small intestine exert a discrete function in pathogen recognition.

In distal parts (jejunum, ileum) of the intestine, several PRRs such as TLR1 to 6, 9, 10, NOD1, NOD2, and RIG-I, and genes associated with PRR signaling pathways IFN*γ*, IL-1*β*, IL-6, IL-10, IL-12, TNF*α,* and TGF*β* are highly expressed. These data, obtained in pig, are in line with those obtained in mouse for TLR2 and TLR4 expression (Ortega-Cava et al. 2003) and in human for TLR-2, -3 and -4 expression (Cario and Podolsky 2000; Otte et al. 2004; Abreu 2010). The high expression of PRRs in the jejunum and the ileum correlates with the high concentration and diversity of microorganisms in these distal segments of the intestine (Wang et al. 2005). Indeed it is well-known that these PAMPs such as TLR4 ligand LPS are shared by many bacteria composing the intestine microbiota (Wang et al. 2005; Lotz et al. 2006). An interaction between these PAMP from Gram-negative bacteria and TLR4 receptor activates then signaling pathways leading to inflammatory cytokine production. These data highlight the key role of distal parts of small intestine in microorganism sensing.

### High expression of PRRs in PP

PRR were differentially expressed between small intestinal segments with and without PP. Indeed, TLR1, 9, and NOD2 showed a higher expression in both jejunal and ileal PP. TLR2, 4, 7, and NOD1 also displayed a differential expression between jejunum and jejunal Peyer's Patches. Immune cells are likely responsible for this specific expression, as these lymphoid tissues are enriched in cell types such as dendritic cells, macrophages, and B and T lymphocytes that express these PRRs (Applequist et al. 2002). These data are in line with the function of Peyer's Patches. These intestinal structures participate more to the recognition of several motifs including lipoproteins, peptidoglycans, and DNA than corresponding intestinal segments. In addition, jejunal Peyer's Patches sample more other PAMPs such as lipoteichoic acid, lipopolysaccharides, single-strand RNA, or mannan than jejunal tissue in general (Kawai and Akira 2001). Peyer's patches possess a special cell type, M cell, which may enable the translocation of bacteria across the epithelial barrier (Abreu 2010). These bacteria may be phagocytosed by macrophages. Bacterial DNA delivered then in the intracellular compartment may be fixed by TLR9 that bound to the inner membrane of intracellular lysosomes (Kawai and Akira 2001). TLR9 activation induces NFkB signaling cascades leading to inflammatory cytokines secretion and immune response induction.

TLR2, 6, 7, 9, 10, and NALP3 differentially expressed in the jejunal and ileal Peyer's Patches. These two gut-associated lymphoid tissues are known to exert distinct immunological function (Butler and Sinkora 2013). For example, ileal Peyer's Patches are less important for B-cell development and maintenance than jejunal Peyer's Patches (Butler and Sinkora 2013). In this study, we also demonstrate that the two Peyer's Patches differ in PRR expression, ileal Peyer's Patches expressing lower levels of TLR7, NOD1, NALP3, RIG-I, as well as IFN*γ*, IL-1 *β*, and IL-12. It is thus possible that jejunal Peyer's Patches recognize most efficiently several patterns including single-strand RNA, double-strand RNA, lipoproteins, or pore-forming toxins (Kawai and Akira 2001; Williams et al. 2010). These results highlight the physiological differences displayed by the two types of Peyer's patches in pigs.

### Low expression of PRR expression in the intestinal crypt

The second part of this study aimed at analyzing the expression of PRRs and cytokines along the crypt–villus axis of the jejunum.

A low level of all PRRs transcripts was observed in the crypts suggesting a moderate involvement of this histological structure in pathogen recognition. Located at the base of the crypts, Paneth cells are known to secrete antimicrobial peptides such as lysozyme (Ouellette 1997). As expected, a high expression of the gene encoding for lysozyme was observed in this fraction. In mice, Paneth cells isolated from the colon were shown to possess high antigen presentation function and to express high levels of TLR9 (Rumio et al. 2004). In contrast, in this study, we observed a poor expression of TLR9, as well as of TLR1, 3, 4, NOD1, and NOD2 in the “crypt” fraction microdissected from pig jejunum. This discrepancy might be due to the species- or the organ-specific expression of TLR9. Presence of Paneth cells in pig jejunum is still controversial (Gonzalez et al. 2013). Thus, lysozyme-producing cells present in pig crypts may not display the same functions than human or mouse Paneth cells. Taken together our data suggest that the jejunal crypts exert a discrete function in pathogen recognition.

### High expression of PRR in *Lamina propria*

Several PRRs including TLR1, 2, 4, 9, NOD1, and NOD2 strongly expressed in the *Lamina propria*. The immune cells and/or professional antigen presenting cells present in this tissue may be responsible for this PRR expression. Indeed, dendritic cells and macrophages are known to express some PRRs such as TLR2, 4, 6, and 9 (Kawai and Akira 2001; Applequist et al. 2002; Gilliet et al. 2008; Reizis et al. 2011). Similarly, Kosik-Bogacka et al. (Kosik-Bogacka et al. 2012) have observed that mouse jejunum possesses TLR2-positive mononuclear cells in the *Lamina propria*. This high expression of PRRs and cytokine in the *Lamina propria* is coherent with the high expression of these genes observed in the MLN. It is notably the case for NOD2 known to be implicated in the innate-adaptive immunity crosstalk that enables T- and B-cell activation (Williams et al. 2010). These PRRs may be expressed by dendritic cells that migrate into mesenteric lymph nodes. Lipoproteins may cross the intestinal epithelial barrier, be internalized by dendritic cells and fixed by NOD2 receptor in cell cytoplasm. The antigen presenting cell may migrate from *Lamina propria* to a mesenteric lymph node where it may secrete inflammatory cytokines and present PAMP to adaptive immune cells.

### High expression of PRR expression in the intestinal villi

Three PRRs, TLR3, 5, and RIG-I, are highly expressed in the epithelium of the villus. It is interesting to note that the same genes were also highly expressed in intestinal segments deprived of lymphoid structures. It can thus be postulated that epithelial cells are responsible for the high expression of these receptors. Primary cultures of epithelial cells from mouse small bowel expressed TLR5 (Choi et al. 2010). TLR3 is highly expressed by terminal ileum epithelial cells in human (Cario and Podolsky 2000). Our results suggest that intestinal epithelial cells also express RIG-I. Among these three PRRs, TLR3, and RIG-I recognize double-strand RNA (Kawai and Akira 2001) and TLR5 recognizes flagellin (Kawai and Akira 2001), two PAMPs linked with pathogenicity (Ramos et al. 2004). The other tested PRRs (TLR1, 2, 4, 6, 7, 8, 9, 10, NOD1, NOD2 and NALP3) were expressed at very low levels in the epithelium. Each of these receptors recognizes PAMPs expressed on different types of microbes. For example, TLR9 ligands are DNA fragments from bacteria, viruses, fungi, and protozoa (Kawai and Akira 2001). The high concentration of the flora-derived PAMPs in the gut lumen might be linked with the low expression level of the genes associated with PRR signaling pathways in the epithelium. Indeed, signaling cascades downstream of TLR can be desensitized by continuous exposure to their targeted PAMPs as described for lipopolysaccharide exposure and TLR4 expression (Lotz et al. 2006). Epithelial cells of villus seem thus to play a crucial role in pathogenic microorganism recognition. They constitute the first sensor that can sample luminal antigens. When an epithelial cell is infected by a virus, RNA viral fragments may be fixed by RNA helicase domain of RIG-I receptor in cytoplasm (Kumar et al. 2011). RIG-I activation leads to type I interferon production that will inhibit viral replication, induce the apoptosis of the cell and activate various component of both innate and adaptive immune response (Kumar et al. 2011).

## Conclusion

This study is the first to analyze the mRNA and protein expression of PRRs and cytokines in the small intestine with a particular emphasis on the variations (2) along the proximal–distal axis, (2) between segments and their relative lymphoid organs, and (3) through crypt–villus axis. The data highlight differences in expression of these molecules in relation to their physiological functions and the cell types of intestinal tissues. Improved knowledge of the expression pattern of PRRs would allow a better understanding of the intestinal sensing of microbiota, maintaining tolerance to commensal microbiota as well as inducing inflammation against pathogens. Because the ligands of PRRs can be used as vaccine adjuvants, their organ and tissue distribution will help in eliciting the desired immune response. This data constitute a baseline to investigate the effect of diet, enteric infection and food contamination on the intestinal responses (Bouhet and Oswald 2007; Pié et al. 2007).
